# A254 CANAGLIFLOZIN EXACERBATES CHEMICALLY INDUCED COLITIS WHEN COMBINED WITH A HIGH SUGAR DIET

**DOI:** 10.1093/jcag/gwad061.254

**Published:** 2024-02-14

**Authors:** K Madsen, T Omeltchenko, N Hotte, A Thiesen, B Villaflor, C Cheng

**Affiliations:** University of Alberta, Edmonton, AB, Canada; University of Alberta, Edmonton, AB, Canada; University of Alberta, Edmonton, AB, Canada; University of Alberta, Edmonton, AB, Canada; University of Alberta, Edmonton, AB, Canada; University of Alberta, Edmonton, AB, Canada

## Abstract

**Background:**

Consumption of high-sugar diets exacerbates inflammation and hinders the healing process in animal models of acute colitis. Canagliflozin (CANA) is a sodium glucose cotransporter-2 (SGLT2) inhibitor primarily used for the management of type 2 diabetes and cardiovascular disease. CANA has demonstrated the ability to suppress human T cell and macrophage effector function and to improve inflammation in rat colitis models. Based on these findings, we hypothesized that CANA would mitigate the deleterious effects of a high sugar diet in a DSS colitis model through its effects on immune effector cells.

**Aims:**

The aim of this study was to examine the effects of a high sugar diet and CANA administration on colonic inflammation in an animal model of acute colitis.

**Methods:**

Adult wild-type male and female 129/SvEv mice were placed on standard chow or a high-sugar diet (HSD) containing 50% sucrose (AIN76A) ± CANA (10mg/kg) gavaged daily (n=4-6 for each group). Following three days on the respective diet, mice received 1.8-2% dextran sodium sulfate (DSS) in their drinking water for 5 days, followed by water alone for 4 days. Disease Activity Index (DAI) was computed daily based on changes in animal weight, stool consistency, and blood in stool. Colonic tissues were evaluated for histological changes, weight-to-length ratio, and cytokine levels using the MesoScale discovery platform.

**Results:**

Mice on the HSD had increased severity of colitis compared with chow fed mice as evidenced by enhanced DAI, increased weight loss, and increased levels of colonic TNFα (pampersand:003C0.02). Response to CANA treatment was both sex and diet dependent. Contrary to our hypothesis, CANA treatment increased severity of colitis in male mice on the HSD as evidenced by increased DAI and delayed healing with increased enterocyte injury and increased levels of lamina propria neutrophils and lymphocytes. CANA treatment did not exacerbate disease severity in females on the HSD but did delay healing as evidenced by increased lamina propria neutrophils and lymphocytes and worse enterocyte injury. These effects were not observed in mice that were fed standard chow diets with CANA.

**Conclusions:**

A high-sugar diet significantly exacerbates chemically induced colitis and delays healing. This effect is intensified when combined with CANA, especially in male mice. These findings indicate that effects of SGLT2 inhibitors on colonic inflammation can be modulated by both dietary intake and sex.

**Funding Agencies:**

CCC

